# Assessing asylum seekers, refugees and undocumented migrants

**DOI:** 10.1192/bjb.2019.67

**Published:** 2020-04

**Authors:** Lauren Z. Waterman, Cara Katona, Cornelius Katona

**Affiliations:** 1 South London and Maudsley NHS Foundation Trust, UK; 2 Camden and Islington Mental Health NHS Trust, UK; 3 Helen Bamber Foundation, UK

**Keywords:** Education and training, refugee, migrant, PTSD, health inequalities

## Abstract

Identifying the causes of psychiatric and physical symptoms in asylum seekers, refugees and other migrants and making definitive diagnoses can be challenging. Ethical and legal challenges in the UK include the likely deterrent effects of upfront charging for National Health Service (NHS) services. This paper focuses on the fictitious case of an asylum seeker presenting to a mental health service in England, highlighting some of the difficulties in assessing and treating this patient group and providing advice to clinicians on clinical and practical management. Current NHS entitlements for migrants are summarised and a list is presented in the online supplement of non-governmental organisations that can provide further support.

## Clinical scenario

You are a psychiatry trainee at an inner-city ‘place of safety’ unit in England. You have been called to see Mr S.T., a 30-year-old Tamil asylum seeker from Sri Lanka, who has been brought in at 22.00 h by police. He is reported to be ‘responding to voices’ and to have collapsed twice, apparently losing consciousness. S.T. speaks minimal English and the Tamil telephone interpreter does not appear to be translating everything he is saying. Furthermore, S.T.'s ability to use the telephone service is limited owing to his distractibility and apparent guardedness towards the interpreter.

S.T. had claimed asylum on arrival at the UK border in 2015. He describes having fled Sri Lanka, where he had been detained and tortured for many months. However, his asylum claim was refused a month ago because of inconsistencies in his account. He was then evicted from accommodation provided by the Home Office and has since been street homeless. The timeline is difficult to establish and his story is not fully coherent.

S.T. states that since his traumatic experiences he has been experiencing frequent visual and auditory hallucinations, mood swings and ‘jumpiness’ whenever there is a loud noise. He avoids being close to anyone wearing a uniform and prefers being alone. He has not had a physical health check since arrival in the UK 4 years ago. He explains that initially he was unaware of how the medical services worked or what he was entitled to. Since his asylum refusal he has been feeling intermittently dizzy and has experienced several episodes of collapse, about which he can remember little. He recently tried to register with a general practitioner (GP); however, the GP practice refused to take him on because he had no proof of address. S.T. stopped trying as he became fearful that the GP would forward his whereabouts to the Home Office and that he would then be sent back to Sri Lanka, where he believes his life would be in danger.

After carrying out an initial assessment, you are uncertain whether there might be an underlying organic cause to S.T.'s presentation, as he has not had any previous medical investigations in the UK. You also feel that he would benefit from a period of in-patient assessment to further clarify the diagnosis and risk. Furthermore, because he is street homeless there is no safe place to discharge him to. He is consenting to in-patient admission but has no funds to pay for National Health Service (NHS) secondary care and you are unsure what NHS care he would be eligible for. The key questions raised by this case are presented in Box 1.

Box 1 Key questions

• What is this person's most likely diagnosis?
• What are the possible causes of his physical symptoms?
• What NHS treatment is he eligible for?
• What management would you provide?
• What political and social issues may you need to consider?

## Assessment, differential diagnosis and formulation

### Could there be a non-psychiatric cause for S.T.’s physical symptoms?

Many migrants are not registered with a GP, for reasons described below. It is therefore common to encounter someone such as S.T. who, despite being at high risk for physical and mental illness, has not previously accessed healthcare since arrival in the UK and may have multiple undiagnosed and untreated illnesses. This adds significant complexity to the diagnostic assessment as, although asylum seekers are more likely to have physical symptoms as a result of psychological trauma, they may also have undiagnosed physical illnesses and physical injuries as a result of torture. It is therefore vital to complete a full physical health workup, including screening for tuberculosis and sexually transmitted infections where indicated, in order to identify and treat medical conditions. Extra care should be taken during physical examinations to avoid retraumatising the patient.

Dizziness and collapse are important features of S.T.'s presentation. Not only does he find these episodes distressing but his lack of awareness during the episodes renders him vulnerable to injury, exploitation and violence. When assessing patients who are in severe distress it is important to be mindful of the potential for diagnostic overshadowing and vital to identify possible medical causes for these episodes. These could include epilepsy, non-epileptic attack disorder, hypoglycaemia, dehydration and vasovagal syncope (fainting), and potentially substance misuse. If a neurological cause is suspected, referral to neurology may be indicated. Epilepsy is often difficult to distinguish from non-epileptic seizures and the two may both occur in the same individual. Important causes of non-epileptic seizures (particularly in people who have been severely traumatised) include panic attacks and dissociative episodes.^1^ Panic attacks are usually rapid but not immediate in onset and are associated with prominent autonomic symptoms such as palpitations, shortness of breath and tingling in the extremities. Dissociation is a psychological defence mechanism manifested as a perceived detachment of the mind from the emotional state and the body. It often occurs in the aftermath of severe trauma and may be triggered by reminders of the trauma. In our clinical experience, episodes of dissociative ‘unconsciousness’ are particularly common in people who have been subjected to sexual trauma and who have an overwhelming sense of shame and revulsion about what has been done to them.

A detailed history, including a collateral history (where possible), neurological examination and simple investigations (such as blood sugar and blood pressure, both during and after the episode) can be helpful to distinguish between these causes.

### Psychotic phenomena in PTSD: a diagnostic challenge

S.T. was reported to be ‘responding to voices’. This might initially be thought to indicate a primary psychotic illness such as schizophrenia. However, it is also important to consider whether the voices might be a manifestation of post-traumatic stress disorder (PTSD). In PTSD, individuals may hear voices as part of a flashback to past traumatic events. Flashbacks may be triggered by reminders of the trauma or stressful situations and could therefore be intensified when the person encounters uniformed officers or is placed in a confined space (such as in hospital). Victims may find it difficult to distinguish these vivid phenomena from reality and may respond to them.

Ways in which flashbacks can be differentiated from primary psychotic experiences include: by their close relationship to the past traumatic experiences; by their transience; and by the preservation of some degree of insight. However, many experts believe that some people with PTSD also have more generalised psychotic experiences which are best understood as part of the PTSD process. This has been termed ‘PTSD with secondary psychotic features’.^2^ A history of trauma is also common in people with psychosis. Therefore, it can be challenging to make a differential diagnosis of a psychotic illness in someone with comorbid symptoms of PTSD and it is common for clinicians to disagree on the primary diagnosis.

### Distinguishing complex PTSD from PTSD and emotionally unstable personality disorder

Complex post-traumatic stress disorder (complex PTSD) is a new diagnosis included in the forthcoming ICD-11.^3^ Complex PTSD is more likely to develop following exposure to prolonged or repeated traumatic events from which escape is difficult (such as torture, slavery, human trafficking, prolonged domestic violence and repeated childhood abuse), whereas PTSD tends to develop following isolated traumatic events.^4^ Complex PTSD is also more likely to develop following interpersonal trauma than following events such as a road-traffic accident.^4^ Complex PTSD shares the same core features of PTSD (exposure to a threatening or horrific event, re-experiencing of the traumatic event, avoidance of traumatic reminders, sense of current threat and interference with functioning) but includes three additional features, all of which must be present for the diagnosis to be made. These are: interpersonal disturbances, affect dysregulation and a persistent negative self-concept. These additional features are believed to result from the degradation of the person's self-identity and autonomy. Functional impairment tends to be worse in complex PTSD than in PTSD, and standard PTSD treatment may be less effective.^4^

It can sometimes be challenging to distinguish between complex PTSD and PTSD with comorbid emotionally unstable personality disorder (EUPD), since complex PTSD and EUPD may both stem from trauma in early life and share disturbances in affect regulation, self-image and interpersonal relationships. Although a pertinent feature of personality disorders is that, by definition, they develop in childhood, it can be particularly difficult to differentiate the disorders in someone who has experienced traumas at an early age or for whom we know little about their premorbid personality, like our patient S.T. However, there are some key differences: in EUPD, the person's self-image and interpersonal relationships tend to be unstable, whereas in complex PTSD the person is more likely to avoid relationships and have a persistently negative self-image.^5^

### Cultural idioms of distress

It is important to consider cultural idioms of distress when assessing patients from different backgrounds. For example, perceptual disturbances such as hearing voices might have a different significance or meaning to that assumed in a Western medical model. As with any symptom, asking the patient what it means to them is often illuminating. The section on cultural formulation in DSM-5 has some useful questions in this regard.^6^

### A challenge for assessment: memory deficits are common following trauma

Trauma and its associated disorders, including PTSD and depression, are associated with relative deficits in autobiographical memory retrieval,^7,8^ and there is evidence that other aspects of memory may also be affected.^9^ As a result, asylum seekers who have experienced trauma may tell a story that is convoluted and has an inconsistent timeline, which can make eliciting a clear history challenging. It is often helpful to check back frequently with the patient to make sure that you have understood correctly and to reflect with the patient on elements of the account that you find inconsistent or incomprehensible. The process of recounting may be distressing for the patient; thus, breaks may need to be taken and it is often not feasible to elicit a full account in a single session.

Of note, it is therefore possible that S.T.'s poor memory had affected his asylum claim, since an inconsistent story or inability to recall specific memories is often considered by immigration systems to indicate poor credibility, despite this link between trauma and memory deficit.^10^

### Decompensation since refusal of the asylum claim

S.T.'s mental health worsened following the refusal of his asylum claim. This is not surprising, since such refusals are often associated with being discredited and disbelieved and with the threat of imminent removal to a place where the individual believes themselves to be unsafe. Refusal of an asylum claim is also often associated with loss of accommodation and financial support. Even for those who are currently awaiting a decision, the research evidence indicates that prolonged immigration uncertainty is associated with a deterioration in mental health.^11^

Many asylum seekers' claims are refused because of inadequate legal representation and/or lack of evidence to support their claim. These individuals may present in crisis and healthcare professionals are often unsure how to help people with such a precarious socio-legal situation. A number of useful charities that provide psychological, social and/or legal support are listed in online supplement 1 (available at https://doi.org/10.1192/bjb.2019.67).

## Eligibility for treatment from NHS primary care, in-patient and community mental health services, and common access barriers

Is it important to note that it is not your job as a clinician to make decisions about who should receive NHS treatment free of charge. This responsibility lies with the NHS trust. According to General Medical Council guidance, the clinician's primary duty is to treat the patient. However, you may be asked by your NHS trust about the clinical urgency of providing treatment for patients who the trust has deemed otherwise ‘ineligible’ for free treatment (as highlighted below). Additionally, it is important to be aware of the eligibility for NHS services of different migrant groups so that, in making your management plan, you have an idea of potential barriers to access for these patients and can advocate for them as necessary.

### Access and barriers to treatment in general practice

#### Eligibility for primary healthcare

According to guidance issued by NHS England in November 2015, anyone in England can register with a GP and receive GP services without charge and ‘GP practices are not required to request any proof of identity or of immigration status from patients wishing to register’.^12^

#### Barriers to registering with a GP

GP practices often mistakenly believe that prospective patients need to provide proof of address and residency, even though that is not legally required. This can result in vulnerable migrants being turned away. For example, of 1717 migrants who approached a charity following difficulties registering with a GP, 20% were still wrongly refused GP access even when supported by a charity case worker.^13^ Some GP practices register migrants as temporary patients,^14^ even though they are eligible to be registered as permanent. Migrants such as S.T. may not have a fixed address or may not have access to identity documents or proof of address.

### Data protection and confidentiality issues

An additional barrier is that refused asylum seekers and undocumented migrants may be afraid to give personal details to a GP practice in case these details are accessed by the Home Office, which could in turn lead them to be arrested, detained and/or deported. Some try to get around this by registering using an alias.^15^ Their fears are well-founded. Non-clinical information about patients may be disclosed to the Home Office by NHS services in certain situations, such as if a patient who is ineligible for free treatment does not pay their treatment bill within 2 months. Their debt to the NHS may also affect their future immigration applications.^12^ Previously, a memorandum of understanding (MoU) stated that NHS Digital could also disclose confidential patient information to the Home Office for the purpose of assisting immigration enforcement.^16^ However, this MoU was withdrawn for amendment in May 2018 and it has not yet been re-released.^12^ At the time of writing, it is unclear how confidential information will be shared with the Home Office in the near future.

### Access and barriers to treatment in secondary care (including community mental healthcare) and in-patient services

#### Asylum seekers, refugees and victims of torture are exempt from NHS charges across all services

All NHS services in England are currently free of charge for asylum seekers (those who have claimed asylum in the UK and are awaiting a decision from the Home Office), those with a rejected asylum/human rights application but who have officially appealed their rejected claim, refugees (those whose asylum claim has been approved) and suspected victims of human trafficking, among a number of other categories.^12^ Also, refused asylum seekers can continue, free of charge, with any course of treatment already underway before their application was refused.^12^ Additionally, a category of NHS services that is currently free of charge irrespective of immigration status is ‘services for the treatment of a physical or mental condition caused by torture, female genital mutilation, domestic violence, or sexual violence’,^12^ which would apply to S.T., whose reported history of undergoing torture may have contributed to his current illness.

However, for those belonging to these exempt categories, the lack of clarity and misinformation about who is eligible for free care has had a deterrent effect and made many vulnerable individuals reluctant to present to services. These individuals often have complex legal situations or are unable to provide the documents requested. Furthermore, administrative staff rarely receive sufficient training in immigration law to adequately determine eligibility for care.^17^

#### New upfront eligibility checks and upfront charging regulations

Following new government regulations introduced in October 2017, all hospital departments in England are legally required to check patients' eligibility for free NHS healthcare. If a patient is unable to prove that they are exempt from charges, they are required to pay upfront in full before receiving any treatment.^18^ This requirement has now been extended to all NHS community health organisations, including community mental health services.^19^ If a patient cannot prove that they are entitled to free care, they have to pay the estimated price for their treatment upfront, unless it is considered ‘urgent’ or ‘immediately necessary’. Doctors will have to review each case to decide whether care is ‘immediately necessary’ or ‘urgent’: if it is deemed immediately necessary/urgent, treatment can be offered and the patient will be charged later; however, any treatment deemed non-urgent can be refused until the patient is able pay upfront.^20^ However, this does not apply to GP care, which is currently free to all, as described above.

#### The effects of upfront charging

Even though the treatment needed is often deemed immediately necessary or urgent, the worry about being charged upfront can deter vulnerable patients from seeking help. A recent analysis of case notes from a Doctors of the World clinic found that 46 patients (over a third of all chargeable cases) had delayed seeking necessary healthcare owing to concerns related to charging, including concerns that their information would be shared with the Home Office.^20^ A number of the UK's medical Royal Colleges have released statements about the upfront charging policy, warning of its risks.^21^

#### What is ‘urgent’ or ‘immediately necessary’ treatment?

There is a lack of clarity from NHS England about what constitutes ‘immediately necessary’ or ‘urgent’ treatment, resulting in confusion and inconsistency between and within services. ‘Immediately necessary’ is usually taken to signify treatment that is life-saving or is needed immediately to prevent a condition from becoming either damaging to the person or life-threatening. Urgent treatment is usually taken to signify treatment that, owing to pain, disability or the risk of the condition worsening without treatment, cannot wait until the person returns to their country of residence (it is usually expected that an undocumented migrant will not return to their home country for at least 6 months).^22^ Treatment is deemed ‘non-urgent’ if ‘it can wait until the patient can reasonably be expected to return to their country of residence’.^12^ Many clinicians and healthcare providers believe that the vast majority of healthcare treatment can be legitimately considered to be at least ‘urgent’, given that most physical and mental health conditions could deteriorate without timely treatment.

#### Treatment under the Mental Health Act and Mental Capacity Act

Those who are detained and/or treated under the Mental Health Act 1983 or Mental Capacity Act 2005 are also exempt from charges for treatment.^12^ Therefore, S.T. would not be charged for his treatment if we decide to detain him.

#### What treatment would S.T. be eligible for without charge?

S.T. is consenting to an informal admission and would not be appropriate for community treatment (as he is street homeless). However, would he be eligible for an informal in-patient admission without charge?

S.T. is not currently legally classified as an asylum seeker as his asylum claim has been rejected and he has not yet launched an appeal. However, it could be argued that he still would be eligible for free voluntary psychiatric treatment, both as an in-patient and in the community, as a victim of torture.

Regardless, he should be eligible for voluntary psychiatric treatment (as an in-patient and in the community) without being charged upfront, on the grounds that the medical team consider his treatment to be ‘immediately necessary’ or ‘urgent’. If he does receive treatment on this basis, it is important to note that he would still get a bill for this after his treatment and, if he is unable to pay that bill, his details could be shared with the Home Office, putting him at risk of being detained or deported.

It is vital that this eligibility is clearly explained to him so that he does not become confused and frightened when the hospital conducts its compulsory eligibility checks.

#### How about if he were in Scotland, Wales or Northern Ireland?

The above guidelines only apply to NHS England. Separate guidelines apply to Scotland^23^, Wales^24^ and Northern Ireland.^25^ In Northern Ireland, the eligibility guidelines are similar to in the UK. Key differences are that in Northern Ireland not all migrants are eligible for free GP care, and refused asylum seekers who have had their asylum claim refused since 2015 have the same entitlements as any other ordinary resident. Undocumented migrants (who do not meet the other exception criteria, such as being a victim of human trafficking) are liable to be charged for GP, inpatient and secondary care, but are not charged for A&E treatment, compulsory detention in hospital or treatment for some infectious diseases and HIV.^25^ In Scotland and Wales, asylum seekers and refused asylum seekers are entitled to free primary and secondary healthcare on the same terms as any other ordinary resident.^23,24^ Please see the relevant guidance for further information.

## Management of S.T.’s case

### Which treatments are effective for someone in such a precarious social situation?

In an acute situation such as S.T.’s, the priority should be to allow the patient to feel as safe and comfortable as possible. Measures should be taken to provide a quiet and private space in which to talk to the patient. Short-term use of benzodiazepines should be (cautiously) considered if the patient is acutely agitated or anxious. If in-patient admission is thought to be indicated, clinicians should be mindful of how an acute in-patient psychiatric ward could be distressing for someone with a history of trauma.

Regarding longer-term management options for S.T., psychological treatments appear to have the greatest benefit in reducing PTSD symptoms.^26^ For example, there is robust evidence supporting the use of narrative exposure therapy (NET).^27^ Although a sense of safety is often considered to be a prerequisite for psychological therapies to be effective, NET (which was developed for use in conflict zones) may be beneficial even for patients whose immigration status and social circumstances remain unstable. The humanising effect of having someone trusted to talk to regularly, in a safe space and without judgement, can be an especially helpful aspect of talking therapies. There is emerging evidence to suggest that arts-based therapies can be effective for those who find it more difficult to express themselves verbally about their trauma.^28^

Psychotropic medication can also be used to treat PTSD-related symptoms. Antidepressants can be helpful in treating depressive symptoms in people who have been severely traumatised: mirtazapine is often used because of its hypnotic effect. Antipsychotic medication (such as quetiapine, which is widely used) can be of benefit, particularly in the context of vivid flashbacks or hallucinations or in the management of persistent anxiety and agitation.^29^

### Practicalities and primary needs

S.T. has been made street homeless since the refusal of his asylum claim. This will also have implications for discharge planning if he is admitted to a psychiatric hospital for treatment. It is important to carry out a comprehensive needs and risks assessment as soon as possible and to generate a care plan in which these needs are prioritised appropriately.

If S.T. launches an appeal against his asylum application rejection, he would become eligible for support such as accommodation and certain other benefits; therefore, providing him with information on non-governmental organisations (NGOs) that provide legal advice may be a priority. A number of NGOs also support asylum seekers by providing therapeutic services, English language courses, social inclusion projects, housing and general advice. For example, NACCOM provides a useful list of charities/services that help destitute migrants across the UK (https://naccom.org.uk/projects/). Further information on some of these NGOs is given in online supplement 1.

Online supplement 2 gives advice on working with and assessing capacity via interpreters, which might help in further assessment of S.T.

It is also important to consider that, even if S.T. has a telephone, he might not have credit with which to make outgoing calls.

### Suggested management plan for S.T.

• Informal psychiatric admission for further assessment and formulation
• Obtain collateral history from friends or relatives if possible (and if S.T. consents to this)
• Thorough physical assessment to identify and treat any non-psychiatric illnesses
• Early and proactive assessment of spectrum of needs: including prioritising housing, finances and referral to relevant charities for psychosocial and legal support
• Consideration of short-term use of benzodiazepines if highly agitated
• Consideration of anti-depressant and/or antipsychotic medication
• Referral for talking therapy with a trauma focus (if available locally or via a charity)

## Conclusions

Diagnosing the causes of psychiatric and physical symptoms in asylum seekers and torture victims and making definitive diagnoses can be challenging. It is often difficult to determine whether psychotic symptoms in this group of patients relate to a primary psychotic disorder or to PTSD; and a new diagnosis of complex PTSD in ICD-11 adds to the pool of diagnostic options. Psychological distress is a common aetiological factor in physical symptoms such as dizziness and chronic pain. However, medical causes should not be excluded without sufficient physical health assessment as migrants may also have undiagnosed and untreated physical illness because of difficulty in accessing medical care.

S.T., the patient discussed in this paper, is fictitious and his case study was constructed to depict a realistic scenario based on our clinical experience of working with refugees and asylum seekers. Although his case may appear to be a particularly complex one, it is very common for migrants to encounter many of the barriers to accessing healthcare highlighted here. This can be very stressful for the healthcare team involved, especially if they are unclear about the frequently changing healthcare access requirements. Charities such as the Health Foundation, Doctors of the World, Medical Justice, Freedom from Torture, Medact and the Helen Bamber Foundation regularly release updated guidance that can be helpful.

There remain many ethical and legal issues that need addressing nationally, including the sharing of patient data between NHS services and the Home Office and the likely deterrent effects of upfront charging for NHS services. An urgent assessment is needed into the impact on vulnerable groups of extending charging into NHS community services. There is often confusion for both patients and healthcare staff about eligibility for free NHS care. This can result in patients who are eligible for free healthcare being denied this care, disengaging from healthcare services or not seeking care in the first place.

## Supplementary material

For supplementary material accompanying this paper visit https://doi.org/10.1192/bjb.2019.67. click here to view supplementary material

## About the authors

**Lauren Waterman** is an ST4 higher trainee in psychiatry at South London and Maudsley NHS Foundation Trust and an Academic Clinical Fellow at the Institute of Psychiatry, Psychology and Neuroscience, King's College London, UK. **Cara Katona** is an ST6 higher trainee at Camden and Islington Mental Health NHS Trust, London, UK. **Cornelius Katona** is Medical Director of the Helen Bamber Foundation, Emeritus Professor of Psychiatry at the University of Kent and Honorary Professor in the Division of Psychiatry of the Faculty of Brain Sciences at University College London, UK.
