# Supplementary material for: Assessing asylum seekers, refugees and undocumented migrants
Source: BJPsych Bull. 2020 Apr;44(2):75–80. doi: 10.1192/bjb.2019.67 (PMC7283125; doi:10.1192/bjb.2019.67)
Supplement: Supplementary file 1 [file S2056469419000676sup.zip › S2056469419000676sup001.docx]

**Additional Issues:**

Working with interpreters

Speaking with patients about sensitive issues via interpreters can be challenging, particularly in cases where the person has experienced interpersonal trauma which can affect their trust of others. Such patients can be especially wary of telephone interpreters and may become suspicious that the interpreter is in fact part of a group that has previously harmed or targeted them. In our experience, they may also be reluctant to disclose experiences that they find shameful to people from their ethnic group, such as a history of being sexually assaulted.

It is preferable to use a face-to-face interpreter where possible. It is also useful to ask the patient about their preferences for the interpreter as some patients may feel safer talking to an interpreter from a particular country or ethnic group, which can sometimes be different from their own if they have previously been harmed by people from their own country. If an interpreter has established rapport with the patient, it is preferable to book that same interpreter again in the future if possible. Using a relative or friend of the patient should be avoided as this could result in inaccurate or false translations, especially if a history of violence or trafficking is being concealed or if there is a conflict of interest. These factors may not be apparent to the clinician. The interpreter should be asked to translate things exactly without trying to explain situations themselves and it is important to allow sufficient extra time for the conversation.

For further useful information on working with interpreters, please see the Doctors of the World website.^1^

Assessing capacity to consent for patients via interpreters

ST has indicated that he is prepared to be admitted to hospital. Because of his confusion and communication difficulties, the question arises as to whether he has the necessary capacity to make this decision. The 2005 Mental Capacity Act clarifies that capacity is time and decision specific. The ‘test’ for capacity has two stages.^2^ Stage one is the question of whether ST has an impairment of, or disturbance, in the functioning of mind or brain (such as PTSD or a psychotic illness. The second stage involves ST’s ability to understand, retain, weigh up and communicate information related to the specific decision in question. In this case it is the decision to be admitted to a psychiatric hospital and treated as an inpatient.

The Mental Capacity Act specifies that capacity should be assumed unless and until its absence can be established, and that all reasonable provision should be made to maximise it. For ST, that would include conducting an assessment via a Tamil interpreter. Capacity assessments can be even more difficult when interpreters are involved. It is therefore important to take time over this; to present relevant information about hospitalization in a clear and simple way; and to obtain verbatim statements for each discrete element of the capacity assessment from the patient, ensuring as little as possible is lost in translation.

References:

1) Doctors of the World. *How to use interpreters effectively.* 2018. <https://migrant.health/resources/how_tos/how-to-use-interpreters-effectively>

2) National Institute for Health and Care Excellence. *Decision-making and mental capacity Clinical guideline CG 108.* 2018. https://www.nice.org.uk/guidance/cg108
