# Supplementary material for: Assessing asylum seekers, refugees and undocumented migrants
Source: BJPsych Bull. 2020 Apr;44(2):75–80. doi: 10.1192/bjb.2019.67 (PMC7283125; doi:10.1192/bjb.2019.67)
Supplement: Supplementary file 1 [file S2056469419000676sup.zip › S2056469419000676sup002.docx]

**List of Organisations Supporting Asylum Seekers, Refugees and Migrants**

Many vulnerable migrants may be distrustful of charitable organisations as they may be unsure of their motives or associations with the Home Office. Therefore, it is important to explain to the patient that charities are non-governmental organisations and can be trusted to keep their details confidential.

There are a number of useful charities that patients can be directed to which provide psychological, social and/or legal support. For example:

- Doctors of the World runs a morning helpline to advise migrants on accessing healthcare and also run a healthcare clinic in Bethnal Green, London.
- Helen Bamber Foundation in London offers a medical advisory service, medico-legal report service, psychological support, welfare and housing support, creative arts and employability skills programmes.
- Praxis Community Projects provides legal advice, general advice and counselling. They are based in Bethnal Green: <http://www.praxis.org.uk/>
- Consonant (formerly Migrants Resource Centre and Asylum Aid) provides free advice and support with housing, health and finances for people based in Westminster. They also provide free English and computer classes around London. Legal advice is chargeable but they can support with applications for legal aid. <https://consonant.org.uk/services/>
- Refugee Council provides destitution services and therapeutic services.
- Family Refugee Support Project in Liverpool provides psychotherapy.
- COMPASS provides counselling in Merseyside.
- Refugee Action provides asylum advice and casework services in London, West Midlands, West Yorkshire and North West.
- Coventry Refugee and Migrant Centre provide general advice, legal advice, therapy and social integration groups in Coventry.
- New Routes Integration runs social and befriending groups in Norwich for adults and children.
- Freedom from Torture provides medico-legal assessments, group therapy and legal services.
- NACCOM provides a useful list of charities/services that help destitute migrants across the UK - <https://naccom.org.uk/projects/>
- **For additional options, please see the pan-UK compendium of resources for professionals created by Liverpool John Moores University, available from:** [**https://www.ljmu.ac.uk/microsites/resources-for-professionals-who-support-asylum-seekers-and-refugees**](https://www.ljmu.ac.uk/microsites/resources-for-professionals-who-support-asylum-seekers-and-refugees)

Specific resources for LGBT Migrants and Asylum Seekers:

- UK Lesbian and Gay Immigration Group provides emotional, psychosocial and legal support for LGBTQ+ asylum seekers: <https://uklgig.org.uk/>
- Microrainbow: Provides safe temporary housing for LGBTQ+ asylum seekers and refugees in the UK: <https://microrainbow.org/>
